# A Comparative Analysis of Pervaporation and Membrane Distillation Techniques for Desalination Utilising the Sweeping Air Methodology with Novel and Economical Pervaporation Membranes

**DOI:** 10.3390/polym15214237

**Published:** 2023-10-27

**Authors:** Nouf F. Al-Harby, Mervette El Batouti, Mahmoud M. Elewa

**Affiliations:** 1 Department of Chemistry, College of Science, Qassim University, Buraidah 51452, Saudi Arabia; 2 Chemistry Department, Faculty of Science, Alexandria University, Alexandria 21526, Egypt; mervette_b@yahoo.com; 3 Arab Academy for Science, Technology and Maritime Transport, Alexandria P.O. Box 1029, Egypt; mahmoud.elewa@aast.edu

**Keywords:** pervaporation, membrane distillation, sweeping air, cellulose acetate, PTFE, desalination

## Abstract

This study used the sweeping air approach to conduct a comparative analysis of pervaporation (PV) and membrane distillation (MD) in the context of desalinating saline/hypersaline water. An experimental setup of the sweeping air arrangement was designed and built at a laboratory size to conduct the research. The desalination process using PV used innovatively designed cellulose acetate (CA) membranes specifically adapted for this purpose. Conversely, in the studies involving MD, hydrophobic polytetrafluoroethylene (PTFE) membranes were utilised. CA membranes were fabricated in our laboratory using the phase inversion approach. The physicochemical characteristics of the membranes were assessed using many methodologies, including FTIR spectroscopy, X-ray diffraction (XRD), scanning electron microscopy (SEM), contact angle measurement, and water uptake analysis. This facilitated a more comprehensive comprehension of the impact of the alkaline treatment on these features. The variables that were examined included the kind of membrane, the pore size of the PTFE membrane, the composition of the casting solution of CA, the concentration of the feed solution, the temperature of the feed, and the temperature of the condenser cooling water. The morphologies of the membranes were examined using SEM. The study’s findings indicated that the use of MD resulted in a greater flow and a remarkable percentage of salt rejection (% SR). Furthermore, it was observed that the flux was positively correlated with the feed temperature, while it exhibited an inverse relationship with the cooling water temperature. Moreover, it was observed that the impact of the pore size of the PTFE membrane on the desalination process was found to be minimal. The most optimal outcomes obtained were 13.35 kg/m^2^ h with a percentage salt rejection (% SR) of 99.86, and 17.96 kg/m^2^ h with a % SR of 99.83 at a temperature of 70 °C, while using MD and PV technologies, respectively. Furthermore, both methods demonstrated the capability to desalinate very salty solutions with a salinity level of up to 160 g/L, thereby yielding potable water in a single step.

## 1. Introduction

The issue of water scarcity is a global challenge that is being experienced by many nations, especially in the Middle East and North Africa (MENA) area [1,2]. In order to tackle this issue, innovative membrane technologies have been suggested as a means to enhance the pace of water production and mitigate the problem of saltwater contamination. Recently, there has been an exploration of MD (membrane distillation) and PV (pervaporation) as potential alternatives to RO. This is because MD and PV use vapour as the permeating species, as opposed to water. The driving force in these two processes is the difference in water vapour partial pressure. Additionally, both methods are dependent on moderate input water temperatures rather than high operating pressures [3]. However, the membranes utilised in the desalination processes, such as MD and PV, must possess a specific arrangement of hydrophobic for MD and hydrophilic properties for PV. Fouling in PV/MD desalination membranes is the undesired build-up of substances on the membrane surface or within its pores, which obstructs the passage of water molecules and negatively affects the membrane’s efficiency. Fouling in the PV/MD desalination processes can be caused by multiple factors and has a substantial impact on process efficiency. The following are common causes of fouling in PV/MD desalination membranes: scaling, organic fouling, inorganic fouling, particulate fouling, biological fouling, vapour permeation and condensation, solute concentration polarisation, temperature polarization, and chemical reactions [4]. In pursuit of this objective, it is acknowledged that hydrophobic membranes are susceptible to significant fouling issues and the development of NaCl crystals on the membrane’s surface, which may further compromise the effectiveness of the separation process. In contrast, hydrophilic membranes used in PV have less susceptibility to fouling. Nevertheless, despite extensive research efforts dedicated to addressing these two challenges, there is currently no evidence of any practical uses for desalination using either MD or PV technologies.

While PV is commercially used for dehydrating solvents and removing organics from solutions, its viability for desalination has been questioned due to lower water flux rates (100–1000 g/m^2^ h) compared to RO and MD (60–70 kg/m^2^ h). However, PV’s advantage lies in its potential for desalination because it deals with highly concentrated liquid mixtures (exceeding 95 wt.% water). This high concentration results in a 20-fold greater water vapour driving force, enabling significant salt rejection and water flow when using a hydrophilic membrane [5,6].

Both PV and MD prefer moderate temperatures compared with other conventional thermal processes, like multi-stage flash (MSF) and multi-effect distillation (MED) [7], making them suitable for renewable energy sources, like solar energy or geothermal energy. These temperatures can be maintained using solar thermal energy storage systems, ensuring continuous operation even during nighttime or adverse weather conditions. Aburabie et al. [8] developed a unique self-heating PV membrane that combines traditional PV separation with interfacial heating of a feed solution using a Joule heater built of carbon nanostructures (CNS) and alginate (CaAlg). Resistive heating, which warms the solution at the feed/membrane interface, is started by applying low voltage to the electrically conductive alginate membrane. PV, particularly with hydrophilic membranes, holds promise for desalination, capitalising on its unique concentration-driven process and compatibility with renewable energy sources.

The application under consideration categorises pervaporation membranes into two types: hydrophilic and hydrophobic [9,10]. Hydrophilic pervaporation membranes are commonly employed in applications involving the selective transport of water through a membrane. These applications include desalination and the removal of small quantities of water from organic solutions. Hydrophobic pervaporation membranes are commonly employed for the separation of trace amounts of organic solvents from aqueous solutions. Nevertheless, the research conducted on the use of this technique in water desalination is scarce, as shown by a restricted number of papers [5,11,12,13,14,15]. The membranes used in prior research on PV brine and seawater desalination included a PVA-based functional layer [16,17,18], a GO-based functional layer [19,20,21], an organic silica/silicate-based functional layer [22], cellulose membranes [23,24,25,26], and others [27,28,29]. The temperature of the feed was a critical parameter as it resulted in an increase in diffusivity and a decrease in viscosity when heated. Nevertheless, the intrinsic permeability of the membrane polymer is of utmost significance [30].

The simplicity of Sweeping Air Pervaporation (SAPV) in comparison to Vacuum Pervaporation (VPV) has been noted, but there is a scarcity of literature documenting the utilisation of sweeping air for separations by pervaporation, notably in the context of desalination. When comparing VPV to SAPV, it is evident that the former incurs significantly higher costs. This is primarily due to the requirement for liquid nitrogen, which is both expensive and poses safety concerns during the condensation step. Additionally, the process of VPV is more intricate in comparison to SAPV. The latter method utilises freely available air and achieves condensation at temperatures ranging from 0 to 2 °C, simply by employing an ice-water bath.

The various types of MD are classified based on the permeate flow configuration. These include Direct Contact Membrane Distillation (DCMD) [31,32], Air Gap Membrane Distillation (AGMD) [33,34], Vacuum Membrane Distillation (VMD) [35,36], and Sweeping Gas Membrane Distillation (SGMD) [37,38]. The permeating channels inside the DCMD modules are occupied with clean water, which undergoes condensation when encountering a cold-water stream. The DCMD technique has undergone substantial investigation and is considered a simple design approach. This technology exhibits significant suitability for several applications, such as saltwater desalination, crystallisation, dye wastewater treatment, and heavy metal ion removal [39,40]. The arrangement of DCMD results in increased heat loss compared to other configurations due to the continuous contact between the feed, membrane, and permeate sides [41]. In order to reduce heat losses, an extra compartment comprising an air gap is included between a chilled condensing plate and a membrane in the design of an AGMD system. The AGMD is generally acknowledged in academic circles as a very energy-efficient design, mostly attributed to its use of gravitational force for the collection of permeate flow. The versatility of this design allows for its use in many circumstances, such as desalination [41]. However, the permeate flow in AGMD is relatively lower than in other configurations. This is primarily attributed to the existence of an air gap between the membrane and the cooling surface. In order to optimise the efficiency of the MD system, two methodologies have been used, namely SGMD and VMD. In the process of SGMD, a cold sweep gas is used as the motive force, but in VMD, vacuum pressure is applied on the permeate side to externally collect the condensate from the system. The use of the SGMD and VMD methodologies offers notable benefits in the separation of azeotropic mixtures, the extraction of volatile organic compounds, the processing of alcoholic solutions, and the recovery of aroma constituents [42,43].

Polymeric hydrophobic membranes possess considerable potential for application in the membrane MD process due to their intrinsic hydrophobic characteristics, ease of production, cost effectiveness, and reduced thermal conductivity within the commercially attainable range of 0.1–0.5 W/m·K [44]. These membranes are typically composed of commercially available materials such as polyethylene (PE), polypropylene (PP), polytetrafluoroethylene (PTFE), and polyvinylidene fluoride (PVDF). Polydimethylsiloxane (PDMS), polyether sulfone (PES), polyetherimide (PEI), polytriazole, cellulose acetate (CA), and polyethylene terephthalate (PET) have also been used as polymer materials in MD, although to a lesser extent due to their partially hydrophobic properties [45,46]. The use of these membranes extends to both hollow fibre and flat sheet configurations [47]. Of the several membranes considered, it can be concluded that PTFE has the highest degree of hydrophobicity. The material exhibits a notable level of chemical resistance and thermal stability. Nevertheless, the processing of this task is a considerable challenge. Common training tactics often include the use of stretching exercises and strength training techniques. PVDF membranes provide very accurate resistance characteristics, yet they readily dissolve in solvents, such as triethyl phosphate (TEP) and dimethylformamide (DMF), when maintained at ambient temperature. In contrast, polypropylene (PP) membranes are often structured by stretching and thermal phase inversion techniques, resulting in exceptional solvent resistance [48]. Polymeric membranes often seen in commercial applications include several types, such as flat sheet membranes, hollow fibre membranes, copolymer flat sheets, and tubular membranes. Inorganic membranes include ceramic-based membranes as well as carbon nanotube-based membranes.

However, the presence of membrane scaling, including both blocking and wetting phenomena, is an inevitable challenge that greatly impedes the durability of the membrane over prolonged MD processes [49,50,51,52,53]. The transit of undiluted water vapour from the feed solution to the permeate leads to an increase in the concentration of the feed. As a result, the formation and adherence of crystals onto the membrane surface occurs, resulting in the scaling of the membrane surface [54,55]. Subsequently, the feed solution is introduced into the holes of the membrane, leading to the formation of crystals inside these pores by the process of water vapour transfer [54,56]. The complete wetting of the membrane orifice by these crystals leads to the transport of the feed solution, rather than only water vapour, across the wetted membrane from the feed side to the permeate side. The aforementioned transfer serves as evidence for the conclusion that the membrane’s lifetime has come to an end [44,57]. Hence, in the context of extended-term MD operations, the issue of membrane scaling has significant importance as the concentration of the feed solution rises. The early identification of possible membrane scaling is a crucial element in extending the durability of the membrane module [58].

The objective of this study is to conduct a comparative analysis of desalination techniques utilising PV and MD. To achieve this, a sweeping air unit has been developed and constructed in our laboratory. The PV experiments will employ innovative hydrophilic cellulose-based membranes, fabricated through the phase inversion method. Conversely, the MD experiments will utilise commercially available PTFE membranes. The physicochemical properties of CA membranes were investigated to assess the impact of alkaline treatment, employing techniques such as FTIR spectroscopy, X-ray diffraction, SEM, contact angle measurement, and water uptake analysis. This study aims to examine many factors and their impact on the flow of product water and its salinity. Additionally, a comparison will be made between the two methodologies, highlighting the significant results obtained from each methodology. This study examined several variables that could potentially impact the productivity and salt rejection percentage of the product water. These variables included the membrane type, the pore size of the PTFE membrane, the composition of the casting solution (CA), the concentration of the feed solution (Ci), the feed temperature, the temperature of the condenser cooling water, and the presence of a support layer beneath the CA membrane. A study of the morphology of the manufactured membrane will be conducted using scanning electron microscopy (SEM).

## 2. Materials and Methods

Cellulose acetate powder (CA) from Panreac, Egypt, acetone (A), cellulose acetate butyrate (CAB) from Kanto Kagaku Co., Ltd. (Tokyo, Japan), cellulose butyrate (CB) from Panreac, Egypt, dimethyl phthalate (DMP) and dimethyl formamide (DMF) from Adwic, Egypt, glycerol (G), maleic anhydride (MA), and sodium chloride from El-Nasr Chemicals, Egypt, and sodium hydroxide from Chemajet, Egypt, were utilised in their original form without any modifications. In the MD studies, PTFE membranes with varying pore sizes (0.1 and 0.45 µm) were used, which were obtained as-is from Sterlitech Corp., WA USA.

### 2.1. Fabrication of PV Membranes and Alkaline Treatment

Cellulose acetate (CA) powder and maleic anhydride (MA) powder were introduced into a glass container with a large opening and a stopper. Subsequently, they were dissolved in a solvent combination consisting of dimethylformamide (DMF), dioxane (D), acetone, and dimethyl phthalate (DMP) in varying weight ratios. The mixture was manually agitated with a glass rod until the full dissolution of the CA occurred. Subsequently, the membrane solution was stored overnight in a securely sealed container to allow for the total elimination of air bubbles. The solution was applied onto a glass plate with a smooth and uniform surface using a casting assembly that included a doctor’s blade. Subsequently, the glass plate with the membrane is subjected to ambient conditions for 30 sec to evaporate solvents from the surface and constitute the rejection layer before being submerged in a tray filled with distilled water for a duration of 60 min. This immersion process facilitated the coagulation of the solution, resulting in a noticeable change in the membrane’s appearance from transparent to white. The glass sheet was gradually separated from the material and immersed in a bath of distilled water, in preparation for the process of deacetylation. The membranes underwent a process of full deacetylation by immersing them for a duration of 8 h in an aqueous alkaline solution containing 5 g/L of sodium hydroxide and 15 g/L of sodium chloride at ambient temperature. The membranes underwent a series of washing steps, which included immersing them in distilled water many times and then separating the liquid from the solid by pouring it out. Subsequently, the membranes were kept in distilled water for future use in the PV studies [59,60]. Table 1 presents the composition of cellulosic membranes used in this study.

### 2.2. CA Membrane Characterisation

#### 2.2.1. Analysis of Contact Angles

The examination of contact angle is an essential component in the investigation of membrane characteristics. The investigation of membrane hydrophilicity was conducted at ambient temperature using a contact angle goniometer (DSA 10-Mk2 model, Data Physics Instruments, Krüss GmbH Germany, Hamburg, Germany). The water contact angle of the samples was determined by using drop shape analysis software (ADVANCE 4.1), with the aim of examining the properties of the water pendant drops. A quantity of 2 microlitres (μL) of Milli-Q water was carefully placed onto the surface of the membrane, with a dispensing rate of 24.8 microlitres per minute (μL/min). The contact angle was evaluated at six discrete locations, and the mean value was identified as the resultant measurement.

#### 2.2.2. Examination of Water Absorption

The methodology used for evaluating the water absorption of the unmodified cellulose acetate (CA) membranes and the membranes treated with alkali was as follows: (i) The specimen underwent a drying procedure in an oven set at a temperature of 105 °C for a duration of one day. (ii) Following this, the desiccated membrane was submerged in Milli-Q water at ambient temperature for a duration of two days, facilitating the attainment of adsorption equilibrium. (iii) Subsequently, the moist membrane (*M_w_*) was delicately wiped with a tissue in order to remove any remaining unabsorbed water, and the membrane was swiftly measured for weight. Finally, the mass of the dried membrane (*M_d_*) was obtained by exposing the membrane to a drying procedure in an oven set at a temperature of 105 °C for a period of 24 h, after which it was weighed. The determination of water absorption by the membranes was conducted using the subsequent equation.

(1) $$Water\;uptake\;\left(\%\right)=\frac{M_{w}-M_{d}}{M_{d}}\times 100$$

#### 2.2.3. The Methodology of X-ray Diffraction (XRD)

In order to examine the changes in the structure of untreated CA/alkali-treated CA membranes after undergoing alkaline treatment, an analysis using X-ray diffraction was conducted. The experimental arrangement consisted of a Philips PW1830 diffractometer that was fitted with a Bragg/Brentano θ–2θ setup. The diffractometer was used with CuKα radiation at 45 kV and 30 mA, using a goniometer circle with a diameter of 173 mm. The experimental scan range was established as 3–75° for Rietveld users, and the duration of the data-gathering operation was one hour.

#### 2.2.4. Analysis Using Fourier-Transform Infrared (FTIR)

The chemical composition of both the pristine cellulose acetate (CA) membrane and the alkali-treated membranes was characterised using attenuated total reflectance-Fourier-transform infrared spectroscopy (ATR-FTIR). The ATR-FTIR instrument utilised for this analysis was the Perkin Elmer Spectrum 100, manufactured in the United States. A minimum of three scans were conducted for each sample within the spectral range of 4000–650 cm^−1^.

#### 2.2.5. Thermogravimetric Analysis (TGA)

TGA is a technique used in several scientific disciplines to study the thermal behaviour of materials. The thermal stability of the virgin cellulose acetate (CA) membranes and the membranes treated with alkali was investigated by the use of a thermo-gravimetric analyser (TGA Q 500, TA instruments, New Castle, DE, USA). The specimens, with weights ranging from 4 to 5 milligrammes, were meticulously placed in the sample pan and exposed to a controlled heating procedure. The temperature was incrementally raised at a pace of 10 degrees Celsius per minute until it reached a final value of 550 degrees Celsius. During the course of this procedure, a continuous stream of nitrogen gas was used at a flow rate of 40 millilitres per minute in order to eliminate any undesirable gases. The resultant change in mass was rigorously quantified and documented.

#### 2.2.6. Examination of Mechanical Characteristics

The mechanical properties, namely the tensile strength and Young’s modulus, of both untreated cellulose acetate (CA) membranes and membranes treated with alkali were evaluated using an Instron 5943 testing machine. The studies were carried out under ambient conditions, namely at room temperature, with a consistent velocity of 2 mm per minute. For each unique membrane type, a minimum of three specimens were measured, and afterwards, an average value was computed.

#### 2.2.7. Morphological Characterisation

Morphological characterisation pertains to the systematic examination and delineation of the observable physical attributes and configurations of a certain thing. The present study entails the examination of the shape. The surface and cross-section morphologies of the pristine and alkali-treated CA membrane were analysed using the Philips XL30 scanning electron microscope (SEM) from the Netherlands. In order to perform a comprehensive study of the unaltered CA membrane, which had a skin thickness of 25 μm, the desiccated samples of the membrane were fractured into smaller segments. The pieces were then attached to the SEM holder. In order to conduct a cross-section analysis, the membranes were subjected to immersion in liquid nitrogen to initiate the freezing process. Subsequently, the membranes were cracked and carefully positioned onto the SEM holders. During the alkali treatment of the membranes, the specimens underwent cutting, leading to dimensions of 4 × 4 cm. Following this, the aforementioned samples were immersed in a sodium hydroxide (NaOH) solution for a period of 8 h. Following that, the specimens were immersed in deionised water for a duration of 60 s for the purpose of removing any remaining sodium hydroxide solution that may be present on the membrane. Subsequently, the treated membrane was fragmented into smaller sections and securely attached to the SEM holder in order to perform surface characterisation. In order to conduct cross-section characterisation, the membranes that were exposed to treatment were immersed in liquid nitrogen and then fragmented. Subsequently, the fractured membranes were securely attached to the SEM holder.

### 2.3. Experimental Setup

**Figure 1:**
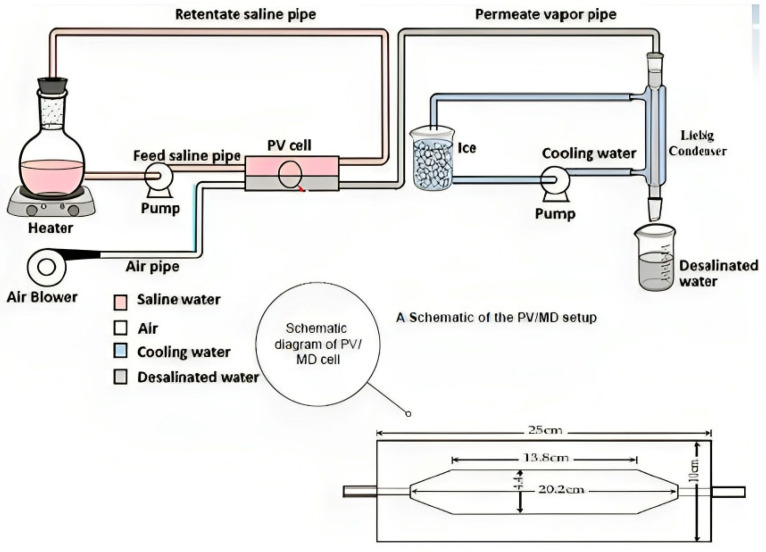
A diagram of the PV/MD arrangement.

The cell was divided into two sections connected by parallel pipes and made of plexiglass. As can be seen in Figure 1, the compartments inside each cell half were hexagonal in shape, measuring 20.2 cm in length, 4.4 cm in width, and 1.5 cm in depth. The membranes were used to entirely divide the two sections of the cell, but before the procedure, they were kept together by a set of six bolts and nuts. Liquid leakage was avoided using rubber gaskets that were strategically positioned in and around the grooves. After the salt solution was forced to flow over the membrane, the cell was clamped horizontally.

Figure 1 depicts a schematic representation of the experimental arrangement, including the PV/MD cell, feed solution flask, electric heater, and saline water pump, which form the heating circuit. Additionally, the cooling circuit is composed of a cooling water pump, a long Liebig condenser, and an ice-water bath. The last path involves the use of purge air, which is supplied by an air blower. This airflow effectively removes the permeating vapour from the downstream area of the cell and directs it through the condenser. Within the condenser, the vapour is condensed into water droplets, which are then gathered in a receiver flask.

The PV/MD membrane was positioned into the test cell, which was thereafter securely sealed using bolts and nuts. The test cell was then oriented horizontally, and the whole assembly was interconnected. A volume of two litres, a solution containing sodium chloride was subjected to heating until it reached the desired temperature. Subsequently, the fluid was allowed to circulate through the upper compartment of the cell. The pump and blower were used to facilitate the operation, during which the condensate was collected at regular intervals of thirty minutes. The collected condensate was then subjected to analysis for concentration using a conductivity metre, while its volume was concurrently measured. The temperature of the solution was also measured at intervals of thirty minutes. The act of recycling the solution served to maintain a consistent temperature in close proximity to the membrane. Each experimental trial was carried out for a minimum duration of six hours, after which the trial was concluded.

### 2.4. PV/MD Membranes Performance

The efficacy of PV/MD membranes is determined by the values of *J* and *% SR*, which are calculated as follows:

(2) $$J=\frac{Q}{A\times t}$$

In this context, *J* represents the flux, *Q* represents the volume of the permeate collected over a specific time period *t*, and *A* represents the effective membrane area.

(3) $$\%\;SR=\frac{\left(C_{i}-C_{f}\right)}{C_{i}}\times 100$$

where *C_i_* and *C_f_* represent the initial and final concentrations of the solution.

## 3. Results and Discussions

### 3.1. CA Membrane Characterisation

#### 3.1.1. Contact Angle

**Figure 2:**
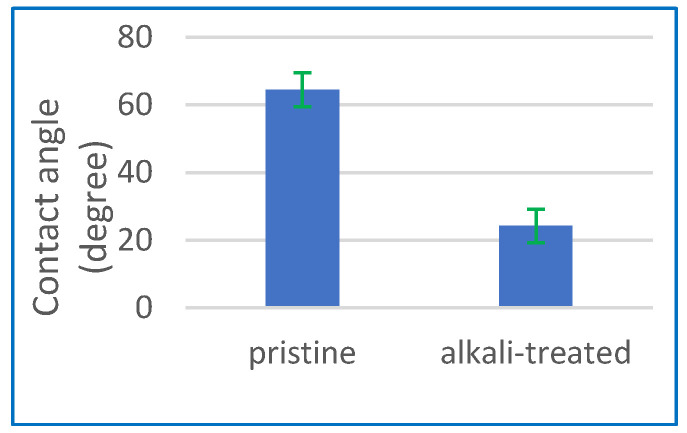
Contact angle for pristine CA membrane and after the alkaline treatment process.

The concept of contact angle refers to the measurement of the angle formed at the interface between a liquid droplet and a solid surface. The characterisation of the enhanced hydrophilicity of the membrane surface after the alkaline treatment was also conducted using contact angle measurement. Figure 2 illustrates a notable reduction in the membrane contact angle, which reduced from an initial value of 64.5° to 24.2° following the alkaline treatment. The observed reduction in contact angle values to 24.2° suggests that the use of alkaline treatment is a very efficient approach for improving the surface hydrophilicity of the CA membranes by introducing hydroxyl groups onto the surface. This can lead to improved wettability and reduced fouling in applications, such as filtration and water treatment.

#### 3.1.2. Measurement of Water Uptake

**Figure 3:**
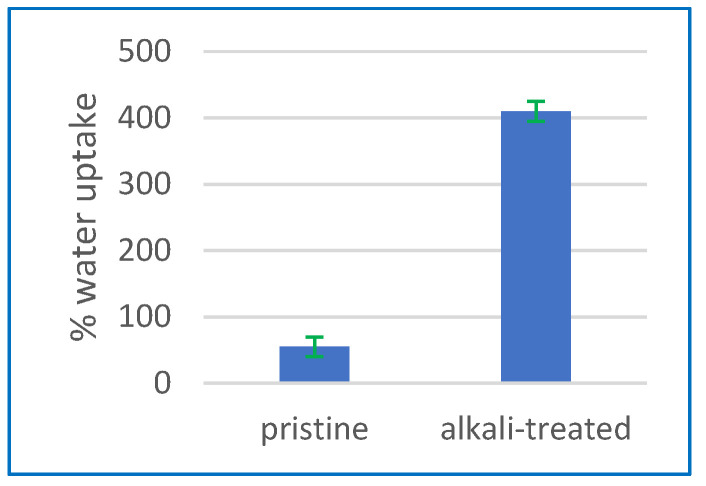
The water absorption characteristics of the membranes both before and after the alkaline treatment procedure.

**Figure 4:**
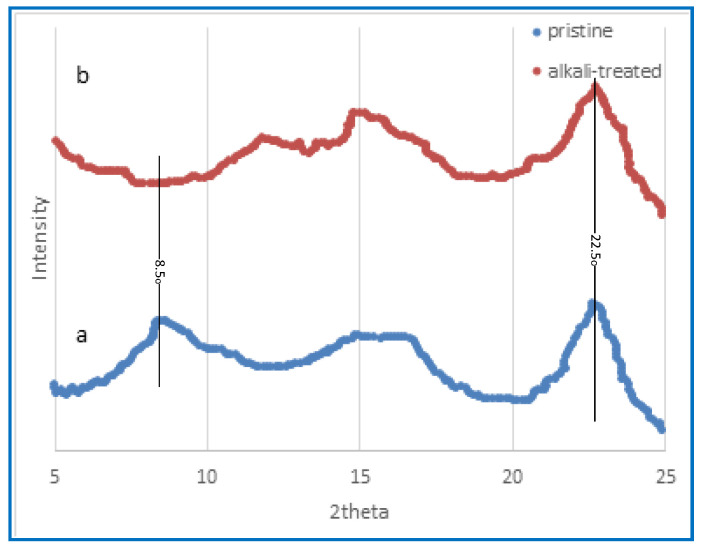
X-ray diffraction pattern obtained from the CA membrane both before and after the alkaline treatment. (**a**) The pattern obtained before treatment; (**b**) the pattern obtained after 8 h of treatment.

The water absorption of the CA membrane was examined by measuring water uptake, with the aim of investigating the impact of alkaline treatment. Figure 3 demonstrates that the absorption of water is greatly increased by treating the CA membrane with an alkaline solution. Figure 4 illustrates a significant increase in water absorption, amounting to a 410% rise, after alkaline treatment in comparison to the membrane that was not subjected to any treatment. The significant increase in water absorption suggests that the alkaline treatment has a beneficial effect on modifying the hydrophilic properties of the CA membrane. The membranes exhibited a higher water absorption as the duration of the alkaline treatment was extended to 24 h. This assertion is further substantiated by the findings from FTIR analysis. The results indicate that an extended duration of alkaline treatment results in the augmentation of O–H groups, contributing to the enhancement of hydrophilicity in the membrane.

#### 3.1.3. X-ray Diffraction

**Figure 5:**
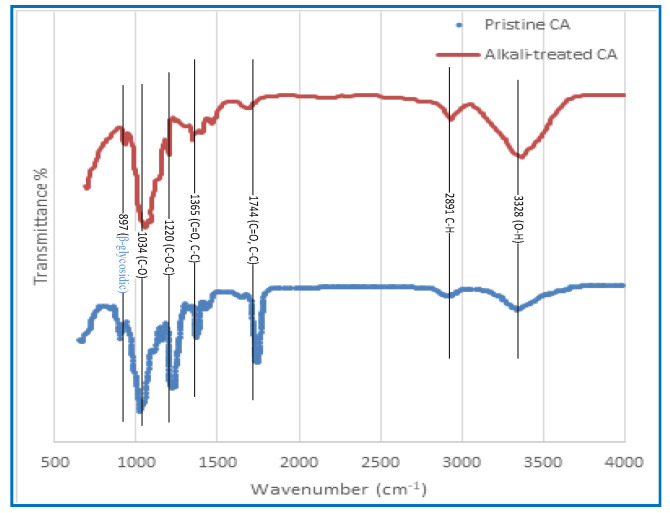
The FTIR spectra of the CA membrane was performed before and after the alkaline treatment.

X-ray diffraction is a scientific technique that involves the scattering of X-rays by a material, which provides valuable information on its atomic and molecular structure. Figure 4 illustrates the X-ray diffraction findings of the membrane composed of CA before and after alkali treatment, specifically focusing on the effects of alkaline treatment. The distinctive peaks associated with cellulose often manifest at around 15°, 22°, and 35°, as reported by Malucelli et al. [61]. The X-ray diffraction (XRD) spectrum of the untreated film membrane, as seen in Figure 5, exhibits the distinctive peak associated with cellulose triacetate within the angular range of 5–10°, which arises from the acetylation process of cellulose [61]. In contrast, the peaks seen in the untreated CA membrane are not present in the alkali-treated CA membrane, as shown in Figure 5. The investigation by Prihatiningtyas et al. [25] focused on analysing the crystalline structure of the CA membrane, using the well-known cellulose crystalline peak at around 22°. They illustrate the reduction in membrane crystalline structure of CTA and CNCs subjected to alkali treatment. Nevertheless, the membrane’s crystalline structure is seen to expand once again when the alkaline treatment duration is extended, commencing at 20 min. The observed rise in levels might be attributed to cellulose recrystallisation, as suggested by Bali et al. [62]. The use of alkaline treatment has been shown to have the capacity to increase both the extent and index of the crystallisation of cellulose [63,64]. This effect is attributed to the lignin elimination from the cellulose structure [64]. In addition, Santos et al. [65] observed that the application of an alkaline treatment using NaOH in hot water at a temperature of 75 °C resulted in an increased crystalline index of the piassava fibres, accompanied by a reduction in the amorphous phase.

#### 3.1.4. Fourier-Transform Infrared (FTIR) Characterisation

The FTIR spectra of untreated CA membranes and CA membranes that have been treated with alkali are shown in Figure 5. A peak can be detected at 3328 cm^−1^, which corresponds to the stretching of O–H bonds, which indicates that the functional groups indicated in Figure 5 are present. In addition, the peak that may be seen at a frequency of 2891 cm^−1^ is related to the stretching of aliphatic C–H bonds. The spectral peaks that can be observed in Figure 5 have been ascribed to the stretching vibrations of the carbon-oxygen double bond (C=O), the carbon–carbon double bond (C=C), and the asymmetric bending of the carbon–hydrogen bond (C–H), respectively [65]. These spectral peaks can be seen at wavenumbers 1744 cm^−1^ and 1365 cm^−1^, respectively. According to [66], the peaks that were seen at 1220 cm^−1^ and 1034 cm1 may be explained by the stretching of the C–O bond and the C–O–C bond in the pyranose ring, respectively. According to Chieng et al. [64] and Santos et al. [65], the existence of glycosidic connections in the glucose ring, which is a hallmark structural property of cellulose, is indicated by the band that was found at 897 cm^−1^. As can be seen in Figure 5, the spectral analysis performed after an alkaline treatment was applied reveals a distinct rising trend in the peak intensity measured at 3328 cm^−1^, which was initially measured at a lower value. The observed trend is significant evidence of an increase in O–H groups as a consequence of the deacetylation process, which involves the replacement of acetate groups with hydroxyl groups. It is possible that the addition of O–H groups is responsible for the observed increase in hydrophilicity of the CA membrane that occurred as a result of the alkaline treatment. In spite of this, the strength of the peak seen at 1220 cm^−1^, which corresponds to the C–O bond, as well as the peak observed at 1744 cm^−1^, which corresponds to the C=O, displayed a notable decline and practically disappeared after the alkaline treatment, mostly as a consequence of the elimination of acetate. Both of these peaks were shown to belong to the C–O bond.

#### 3.1.5. Thermogravimetric Analysis (TGA)

**Figure 6:**
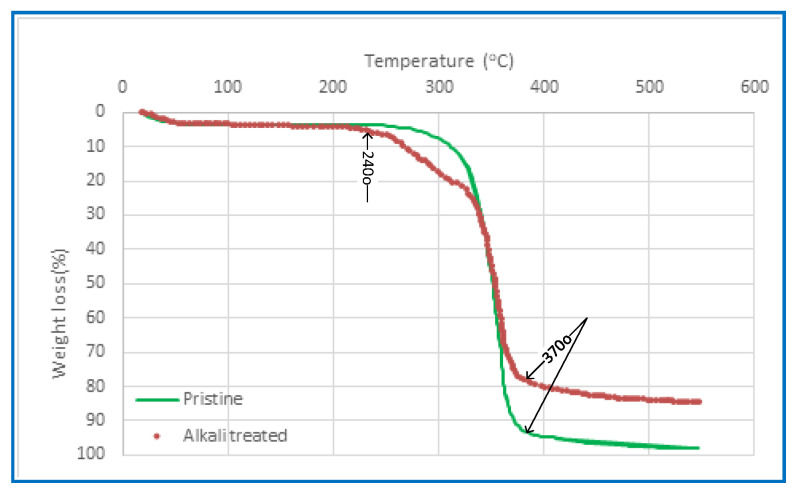
A TGA was performed on the CA membrane both before and after the alkaline treatment.

Thermal stability is a crucial consideration for the PV membrane due to the thermally induced nature of PV separation. The thermal characteristics of CA membranes, which were synthesised using various solvents, were examined before and after alkali treatment via the use of Thermal Gravimetric Analysis (TGA), as seen in Figure 6. The findings indicate a marginal disparity among the TGA curves of the membranes fabricated in the present investigation, whereas the unaltered CA membrane exhibits a significantly distinct behaviour. The decrease in weight seen at temperatures lower than 240 °C is attributed to the process of evaporation, namely the removal of any remaining moisture or solvent. The CA polymer chain’s thermal breakdown occurs within a temperature range of 240–370 °C. The carbonisation and breakdown processes of the membranes occur at temperatures above 370 °C. The residue yield of the CA membrane exhibits an increase compared to the pristine CA membrane. This is particularly relevant considering that PV systems typically operate within a feed temperature range of 30 to 90 °C.

#### 3.1.6. The Mechanical Properties

**Figure 7:**
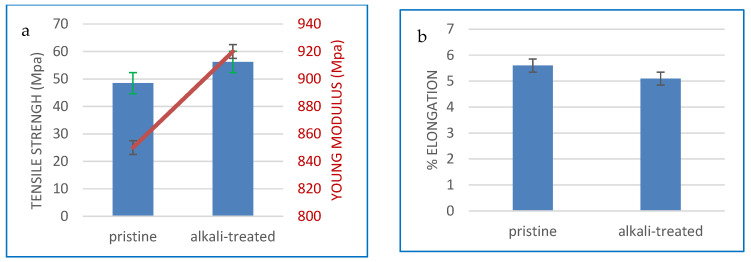
The mechanical characteristics of pristine CA membrane and alkali-treated CA membranes: (**a**) tensile strength and Young’s modulus (**b**) % elongation.

The investigation focused on the analysis of tensile strength, Young’s modulus, and elongation, and the corresponding outcomes are shown in Figure 7. Figure 7a illustrates that the alkaline treatment led to a marginal improvement in mechanical characteristics compared to the original CA membrane, with the values increasing from 48.5 to 56.2 MPa in tensile strength and from 920 to 850 MPa in Young’s modulus.

However, the CA membrane enhances its tensile strength while concurrently reducing its elongation at break (Figure 7b). The alkali-treated CA membranes exhibit increased hardness and reduced deformability as a result of fractures occurring at low strain levels. The manipulation of solvent concentrations within the CA dope solution has a discernible impact on the quality of dispersion, leading to the formation of macro-and/or micro-voids within the membrane structure. Consequently, the membrane becomes susceptible to fragility.

#### 3.1.7. Morphological Characterisation

**Figure 8:**
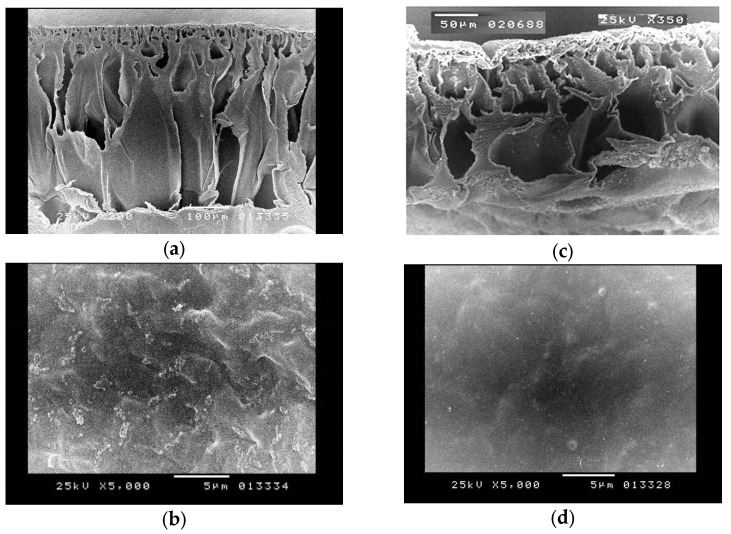
SEM for a pristine CA membrane: (**a**) cross-section, (**b**) surface and treated CA, (**c**) cross-section, (**d**) surface.

The present study aims to provide a comprehensive morphological characterisation of the subject under investigation. Figure 8 presents the scanning electron microscopy (SEM) pictures that depict the surface morphology of the untreated and alkali-treated CA pervaporation membrane. The data shown in the figures indicate that both the pristine and alkali-treated membranes have a compact structure. Figure 8 represents the untreated and alkali-treated CA membranes. Figure 8a,b demonstrate that the untreated CA nanocomposite exhibits a dry and hard state. Conversely, Figure 8c,d illustrate that the CA membrane, after an 8 h treatment in a 5% NaOH solution, becomes moist and squishy.

The cross-section shape of the CA membrane is similarly influenced by the alkaline treatment, as seen in Figure 8. This suggests that the alkali process is not just confined to the surface of the membrane but also takes place inside its internal structure. Furthermore, the structure becomes even more compact and dense after being subjected to alkaline treatment, as illustrated in Figure 8c. The deacetylation reaction is a hydrolysis reaction, which implies that it is catalysed by water. The alkaline solution deprotonates the hydroxyl groups on the glucose units, increasing their reactivity. The hydroxide ions then separate the acetyl groups from the glucose molecules. The deacetylation process produces cellulose, which is a more crystalline substance than CA. The enhanced crystallinity of the cellulose membrane makes it more compact. The main purpose of compacting the structure through alkaline treatment is to alter the physical and chemical characteristics of the CA membrane by improving the crystallinity of the membrane. Alkaline treatment for cellulose acetate membranes creates a compact structure. This is due to the fact that the alkaline treatment removes the acetyl groups from the cellulose acetate, allowing the cellulose chains to pack closer together. This treatment improves the membrane’s performance in desalination by enhancing hydrophilicity, increasing mechanical strength, removing impurities, enhancing chemical stability, and improving selectivity. Following an 8 h exposure to an alkali solution, the membrane pores exhibit signs of collapse as a result of the deacetylation process, aligning with findings reported in the literature [67].

### 3.2. PTFE Membrane Properties

**Figure 9:**
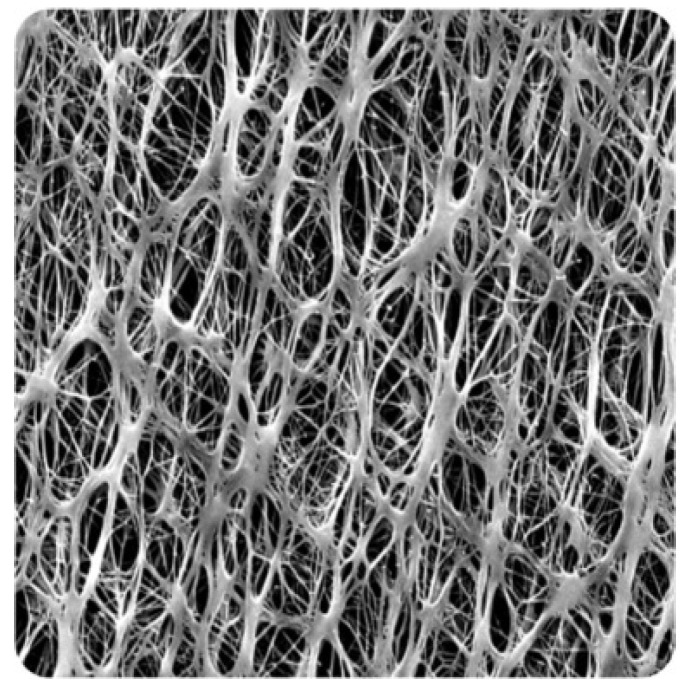
SEM image of a PTFE membrane.

PTFE is a substance with a high hydrophobicity, or resistance to water, which also has a low water absorption rate. The fluorine atoms in PTFE’s molecular structure form a tight connection with the carbon atoms and contribute to the substance’s high non-polarity and lack of reactivity. Because of this, PTFE does not readily absorb or take up water; this means that under typical circumstances, PTFE membranes have very little water absorption, if any at all. Due to its non-stick and water-repellent characteristics, PTFE tends for water droplets to bead up and roll off the surface. This quality qualifies PTFE for uses where water resistance is crucial, including waterproof coatings, non-stick cookware, and several industrial and laboratory settings. Table 2 presents the properties of PTFE at pore sizes of 0.1- and 0.45-micron membranes, which were used in this study and were received by the manufacturer (Sterliteck Corp). Figure 9 depicts the surface of the new membranes, highlighting the PTFE fibrous structure.

### 3.3. CA and PTFE Membrane Performance in PV/MD Desalination

Table 3 displays the experimental conditions and outcomes obtained via the implementation of both methodologies. The membranes used in the PV tests were cellulose butyrate (CB), cellulose acetate butyrate (CAB), and cellulose acetate (CA). Conversely, in the membrane distillation (MD) research, polytetrafluoroethylene (PTFE) membranes with different pore sizes were utilised.

Various parameters were examined to determine their impact on the flow and salinity of the resulting product water. As previously stated, the concept of contact angle refers to the measurement of the angle formed at the interface between a liquid droplet and a solid surface. The characterisation of the enhanced hydrophilicity of the membrane surface after the alkaline treatment was also conducted using contact angle measurement. Figure 2 illustrates a notable reduction in the membrane contact angle, which reduced from an initial value of 64.5° to 24.2° following the alkaline treatment. The observed reduction in contact angle values to 24.2° suggests that the use of alkaline treatment is a very efficient approach for improving the surface hydrophilicity of the CA membranes by introducing hydroxyl groups onto the surface. This can lead to improved wettability and reduced fouling in applications, such as filtration and water treatment. All the experiments were run at least three times regarding the flux and % SR. To determine the standard deviation of multiple flux and % SR measurements conducted under comparable conditions, the necessary calculations were performed, and the error bars can indicate the range of values that extend one standard deviation above and below the mean value.

The use of a CB membrane as the membrane polymer for PV was found to be unsatisfactory due to its low salt rejection capability, although it exhibited a high flux rate. In addition, it is worth noting that the membrane exhibited a lack of strength and experienced tearing during a very short period of operation. Consequently, the membrane was discarded and was not exposed to any further research. The lack of uniformity in the CAB membrane may likely be attributed to the coexistence of the butyrate radical and the acetyl ester group. Consequently, the membrane exhibited streaks and was deemed inappropriate for further PV testing, leading to its exclusion from further analyses. In the case of cellulose acetate (CA) membranes, where CA served as the membrane matrix and the concentration of solute (Ci) was set at an unusually high value of 160 g/L, the observed flux was found to be satisfactory at 10.62 kg/m^2^ h, while the salt rejection percentage (SR %) exhibited a remarkable value of 99.83. The exceptional outcome may be ascribed to two factors. The first one is the well-known fact that CA produces durable and uniform membranes, which has led to its extensive use in high-pressure reverse osmosis (RO) desalination for many years. Furthermore, it should be noted that the formulation of the casting solution was previously optimised, as mentioned in the experimental section, resulting in the production of a highly efficient PV membrane. It is important to acknowledge that the fabrication of this membrane included the process of casting the dope over a polypropylene screen with a specific mesh number. This was performed to provide support to the membrane while ensuring that its performance remained unaffected. Figure 9 illustrates the impact of various cellulosic membranes on the flow and selectivity ratio (SR %) in the context of pervaporation (PV) at a temperature of 70 degrees Celsius.

In the next section, we will discuss desalination by means of sweeping air MD. Table 2 displays the outcomes of using PTFE membranes in a variety of settings. Experiments showed that both the 0.1- and 0.45-micron membranes generated identical salt rejection compared to seawater, demonstrating that MD using this membrane is appropriate for the desalination of hypersaline water, similar to PV with the CA membrane. It is important to note, however, that hydrophobic membranes tend to foul more quickly after extended use than hydrophilic membranes.

Using PTFE membranes with a pore size of 0.45 microns and varying temperatures, it was discovered that a very high flux (13.4 kg/m^2^ h) and outstanding salt rejection (99.86%) could be achieved at a temperature of 70 degrees Celsius. However, when the input temperature was reduced to 60 °C, the flux dropped to 12.18 kg/m^2^ h, and the salt rejection decreased to 98.78%. Given that MD performs best at temperatures around 65 °C, this finding implies that solar heat might be employed advantageously to heat the feed during operation. Additionally, it is understood that operating at a higher temperature (80 °C) may have triggered membrane wetting.

Salt rejection remained substantial and the flux declined, perhaps as a result of partial pore plugging, in PTFE membranes when Ci was raised to 96 g/L and T_f_ was 60 °C, resulting in a flux drop to 8.88 kg/m^2^ h. In the next experiment, Ci was raised to 101.5 g/L, and the flux was, predictably, reduced to 7.99 kg/m^2^ h. As in the previous tests, the % SR remained high (99.73).

#### 3.3.1. The Influence of Input Concentration on Flow and %Salt Rejection

**Figure 10:**
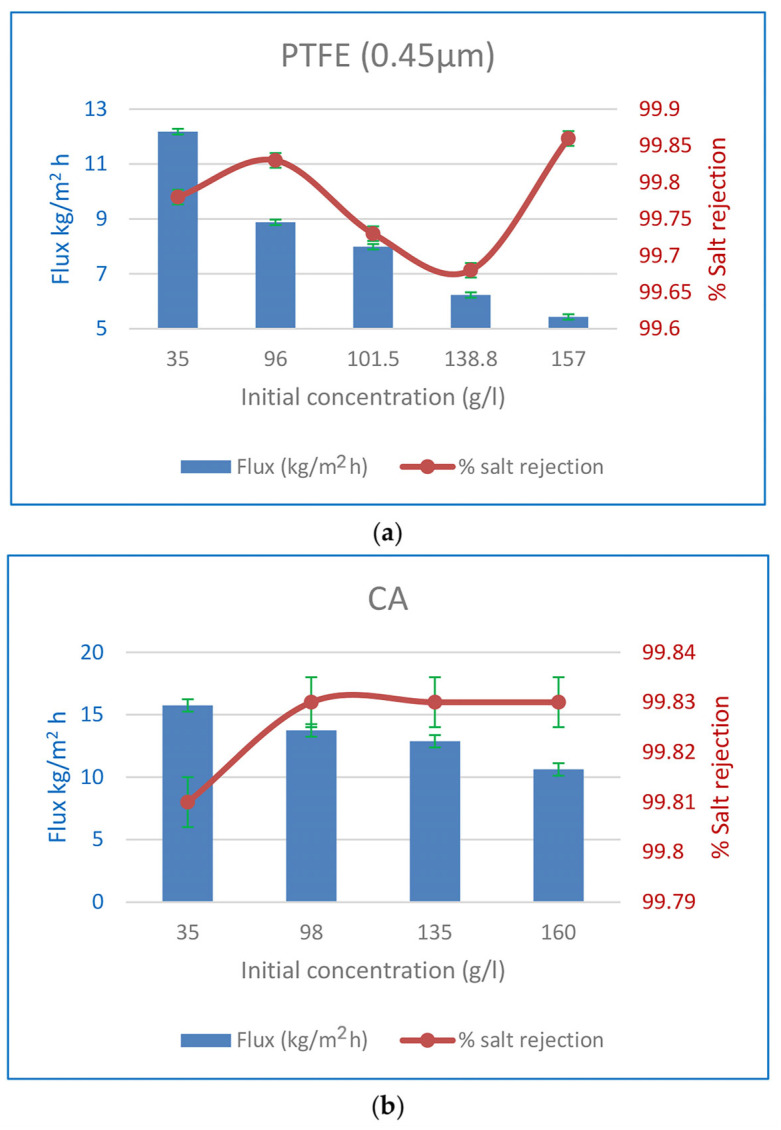
(**a**) Effect of initial feed concentration on flux and %SR for 0.45 µm PTFE MD membranes at 60 °C. (**b**) Effect of initial feed concentration on flux and %SR for CA PV membranes at 60 °C.

Experiments were performed to illustrate the impact of initial feed concentration on the % SR when 0.45 m PTFE membranes were utilised, as shown in Figure 10. At 60 °C, the flow was found to drop from 12.18 to 5.43 kg/m^2^ h when the Ci was raised from 35 to 157 g/L, and from 15.74 to 10.62 kg/m^2^ h when the Ci was increased from 35 to 160 g/L for PV CA membranes operating at the same temperature. The SR percentage never dropped below 99.8%. Increased NaCl content in the feed solution decreases water concentration, which in turn decreases water sorption at the membrane interface and thus decreases water flux [68]. According to Fick’s rule, a rise in the salt feed concentration decreases the thermodynamic activity of water, which in turn reduces the water flux [15]. The feed solution’s water content drops from 96.5 to 84 wt% when salt is increased from 35 to 160 g/L. As a result, water sorption decreases at the liquid membrane contact due to the lower water concentration [24]. The energy needed to propel water molecules across the membrane is less. Increased feed salt causes a rise in the concentration of hydrated ions on the membrane’s surface, which in turn reduces water flow. The effectiveness of the CA PV membrane over a variety of feed solution concentrations (35–160 g/L) was investigated. This concentration range is common and above that of seawater and produced water, although it is still representative. Water flux was reduced by 37% (8.88 kg/m^2^ h) and 15% (13.74 kg/m^2^ h) in PTFE MD and CA PV membranes, respectively, when the salt content was increased to 96 and 98 g/L, although the SR was maintained above 99.8%. This remark suggests that PTFE and CA membranes have desirable properties for the desalination of hypersaline fluids using MD and PV. These membranes may find use in membrane crystallisation processes that attempt to achieve zero liquid discharge (ZLD) by creating purified water and salt crystals from reverse osmosis (RO) brines.

Meng et al. [69] also observed a similar trend, showing that the incompatibility between a hydrophilic polymer, PVA, and a hydrophobic PTFE porous support can be resolved using a straightforward and reliable method called spray coating to produce a membrane suitable for MD or PV. The spray-coated PVA/PTFE membrane achieved a water flux of 64.2 kg/m^2^ h and exhibited a salt rejection rate exceeding 99.9% in DCMD mode. These results were obtained at a feed temperature of 75 °C. The PVA/PTFE composite membranes exhibited a water flux of 143.4 kg/m^2^ h at a temperature of 75 °C in PV mode.

#### 3.3.2. Flux and SR % Affects Due to Feed Temperature

**Figure 11:**
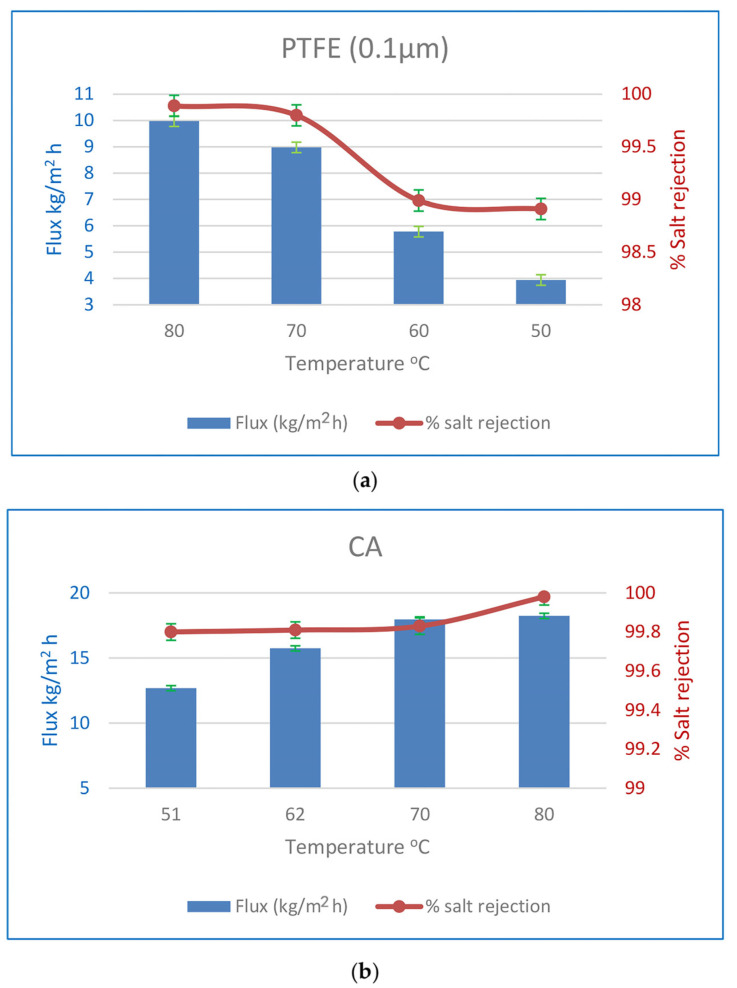
(**a**) Effect of initial feed temperature on flux and % SR for 0.1µm PTFE MD membranes at an initial concentration of 35 g/L. (**b**) Effect of initial feed temperature on flux and % SR for CA PV membranes at an initial concentration of 35 g/L.

Figure 11 shows the impact of feed temperature on flow and SR % in a few selected experiments when a PTFE membrane of 0.1 µm thickness is utilised for MD. Increasing the feed temperature by 30 degrees results in a 2.5-fold increase in flux and an improvement in the SR %. For the water vapour partial pressure difference, the presumed driving power of the process also increases. Due to the hydrophilic nature of the CA membranes and the rise in water vapour partial pressure difference, the flow of CA membranes rose by 43% under the same operating circumstances, with a very minor improvement in the % SR. Feed temperatures that are raised cause a greater water flux rate. Water vapour pressure on the feed side increases exponentially with feed temperature, as a preliminary observation. On the permeate side, the vapour pressure is constant. The water flow increased because the driving power increased. In addition, an increase in temperature increases the water’s diffusion coefficient, making it easier for water molecules to move over the membrane. At higher feed temperatures, the polymeric membrane has a larger free volume, which aids in water molecule diffusion via this space [23,30].

It is clear that when feed temperature rises, the impact of higher salt content on water flow increases. The fundamental contributor to the observed phenomena is the exponential connection between water vapour pressure and temperature. The effect of feed concentration on water’s partial vapour pressure also grows more apparent with increasing feed temperature [15]. As the concentration of the feed increases, the water flow slows significantly.

The link between water flux and operational temperature may be theoretically elucidated via the use of the the Arrhenius equation [70,71,72].

(4) $$J=J_{o\;}exp\left(\frac{{-E}_{a}}{RT}\right)$$

**Figure 12:**
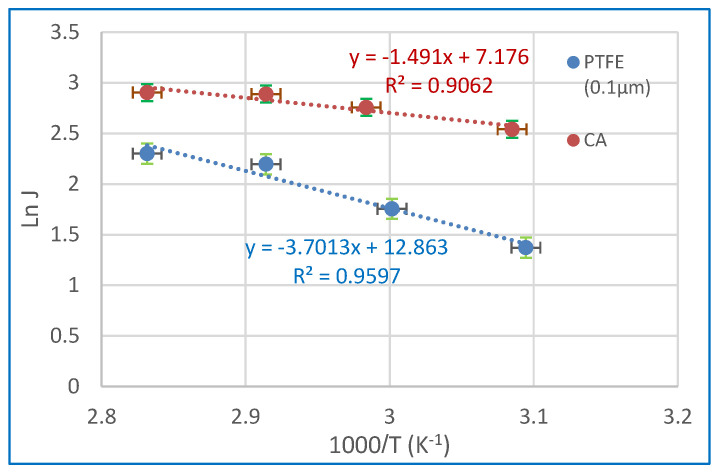
Arrhenius plot of ln (J_o_) and 1000/T of CA and 0.1 µm PTFE membrane at a 35 g/L salt solution.

In the above equation, J represents the water flux (expressed in kg m^−2^ h^−1^), *J_o_* denotes the pre-exponential factor (expressed in kg/m^2^ h), *R* represents the gas constant (expressed in kJ/mol .K), *T* represents the feed temperature (expressed in Kelvin), and *E_a_* represents the activation energy (expressed in kJ/mol). The value of *E_a_* may be calculated by calculating the slope of the Arrhenius plot, which involves plotting the natural logarithm of J against the reciprocal of T (Figure 12). A decrease in perceived activation energy corresponds to increased ease of diffusion for the permeate component across the membrane. Hence, the assessment of membrane materials may be conducted by using the value of E_a_ under identical operational conditions [21]. The activation energy for water is determined to be 12.4 kJ/mol for CA membranes and 30.8 kJ/mol for 0.1 µm PTFE membranes. The activation energy being positive indicates that feed-side water flow rises with increasing temperature [73,74].

#### 3.3.3. Compare Used Membranes with Others Cited

Table 4 contrasts the alkali-treated CA membrane’s desalination performance with the very significant results from other polymer-based membranes published in the literature. The CA membrane demonstrated improved water flux, particularly when desalinating feeds with high salinities reaching 160 g/L NaCl. The membrane features an asymmetrical structure that competes for high water flux. It is worth noting that the activation energy of the fabricated CA is one of the lowest compared to the others reported. The cost-effective CA was fabricated by a simple, well-known phase inversion technique, while most of the others were fabricated via sophisticated techniques with higher-cost materials.

This comparison focuses on the development of high-performance membranes for the use of pervaporation desalination. It has been determined that polyvinyl alcohol (PVA) is a very suitable membrane polymer for desalination by PV. This is mostly due to its exceptional hydrophilicity, which may be ascribed to the abundant presence of hydroxyl groups. Additionally, PVA exhibits stability when combined with filler material and cross-linker components. Currently, the most notable achievements in desalination involve PVA-based thin membranes, which have demonstrated water permeation rates ranging from 143 to 234 kg/m^2^ h. However, there has been recent progress in the development of ultra-thin membranes, measuring less than 100 nm, which incorporate two-dimensional materials, like GO and MXene. These ultra-thin membranes have shown competitive water permeation rates of approximately 85 to 98 kg/m^2^ h, surpassing the performance of state-of-the-art PV membranes [75]. Currently, chemically synthesised polymers need to be replaced. Biopolymers should be used to make environmentally safe membranes because they are cheap, non-toxic, biodegradable, and compatible with living things [76]. So far, only a few biopolymers (like chitosan, cellulose acetate, etc.) have been studied in pervaporation distillation and shown to work well for both absorption and separation. Bio-based polymers could be looked into and pushed as an option to meet the growing demand for membrane materials in the European Union because of the new circular economy [77]. Table 4 also compares the CA membrane’s desalination performance to other PV cellulose-based membranes. According to Table 4, the CA membrane pervaporates better than the membranes employed in previous experiments. Although the manufactured CA was thicker than the other cellulosic membranes, PV desalination achieved a larger water flow. Solution diffusion theory, represented by Fick’s law, states that species flowing across the membrane are counterintuitively proportional to membrane thickness.

It is important to note that the impact of alkali treatment on cellulose acetate membranes can vary depending on the specific treatment conditions, including the type and concentration of the alkali solution, treatment duration, and temperature. Researchers often explore different treatment parameters to optimise membrane properties for specific applications, aiming to balance selectivity and flux.

**Table 4:** Performance evaluation of several PV membranes used to treat a brine solution.

Membrane Name	Thickness (nm)	Contact Angle (°)	Mem Config	PV Config	Feed NaCl (wt %)	Feed Temp (°C)	Activation Energy (kJ mol^−1^)	Flux (kg/m^2^ h)	SR (%)	Ref.
PVA	730 **	n/a	FS	VPV	3.5	75	n/a	234.9	99.7	[78]
PVA/PTFE	2600 *	n/a	FS	VPV	3.5	75	n/a	143.4	99.9	[69]
MXene/PAN	∼60 *	49.5	FS	VPV	3.5	65	13.55	85.4	99.5	[79]
MXene140	∼100 *	52.5	FS	VPV	10	70	32.85	70	99.90	[80]
HPAN-2	148,600 **	34	FS	VPV	10	65	20.1	58.7	99.91	[81]
CTA/CNCs	10,000 **	24.7	FS	VPV	10	70	37.8	58.5	99.8	[25]
PA/mCNT	160 *	62	FS	VPV	10	70	36.97	40.8	99.99	[82]
PVA-Lap2 MMM	120,000–160,000 **	82	FS	VPV	10	70	12.24	39.9	>99.9	[83]
CS/GO-1MMM	10,000–13,000 **	79	FS	VPV	5	81	31.28	30.0	99.99	[84]
Laponite/PVA	120,000–160,000 **	n/a	FS	VPV	10	40	5.73	21.1	99.99	[85]
GO-PI	1,000,000 **	59	HF	VPV	3.5	90	18.7	15.6	>99.8	[86]
GO/PAN	100 *	n/a	FS	VPV	10	30	22.19	11.23	99.8	[87]
S-PVA/PAN	4900 **	77.1	FS	VPV	10	70	n/a	11.2	99.8	[88]
Polyimide/graphene oxide	100,000 **	59	FS	VPV	10	75	15.7	10.7	99.9	[89]
PVA/silica	220 **	n/a	HF	VPV	3	60	14.44	10.4	99.9	[21]
Cellulose diacetate on PTFE	3500 **	n/a	FS	VPV	4	40	n/a	4.5–5.1	100	[11]
Cellulose acetate	20,000–25,000 *	62	FS	SGPV	4–14	70		5.97–3.45	99.7	[60]
Alkali-treated cellulose acetate	25,000 *	24	FS	SGPV	3.5–16	70	12.4	17.96–11.08	>99.8	This study
Cellulose triacetate/Al_2_O_3_	13,000 **	52	HF	VPV	3–9	70	33.3–34.4	6.8–5	99.8	[24]

PAN: polyacrylonitrile; GO: graphene oxide; Lap: laponite; MMM: mixed matrix membrane PVA: polyvinyl alcohol; CS: chitosan; GA: glutaraldehyde; CTA: cellulose triacetate; CNCs: cellulose nanocrystals; PA: polyamide; mCNTs: modified carbon nanotubes; HPAN: hydrolysed polyacrylonitrile; PI: polyimide; GOQDs: graphene oxide quantum dots; * thickness of selective layer; ** thickness of membrane; n/a: not available.

Table 5 PTFE membranes with different thicknesses showed good performance for hypersaline water desalination. PTFE’s strong hydrophobicity, chemical and thermal stability, and oxidation resistance make it an excellent MD membrane material; however, the material is insoluble in common solvents and challenging to electrospin [90,91]. Due to the solvent resistance and melting temperature of PTFE resin, it is not feasible to produce a porous PTFE membrane using conventional phase inversion or melt-spinning techniques, specifically for a porous hollow fibre membrane [92,93]. Other membranes presented in Table 5 were fabricated via the electrospinning technique.

## 4. Conclusions

MD and PV are excellent for unconventional desalination because they manage variable feed salinity effectively. They are more environmentally sustainable since they can use low-grade heat and renewable energy sources. PV technology, especially when using certain membrane materials, may give greater flux than MD but equivalent salt rejections. In this study, the highest achieved outcomes were 13.35 kg/m^2^ h with an SR of 99.86% and 17.96 kg/m^2^ h with a salt rejection rate of 99.83% at a temperature of 70 °C using MD and PV technologies, respectively. Both methods effectively desalinated highly saline solutions with a salinity level of up to 160 g/L, producing drinkable water in a single step. Regarding the CA membrane characterisation, the contact angle values decreased to 24.2°, indicating that alkaline treatment effectively enhances the hydrophilicity of CA membranes by introducing hydroxyl groups onto the surface. Enhanced wettability and decreased fouling can be achieved in various applications, including desalination and water treatment. The water absorption significantly increased by 410% following alkaline treatment compared to the untreated membrane. XRD reveals that alkaline treatment has been demonstrated to enhance the degree and rate of cellulose crystallisation. FTIR confirmed an increase in O–H groups, which is responsible for the hydrophilicity increase at the expense of acetate groups observed in the CA membrane that occurred as a result of the alkaline treatment. TGA suggests that PV systems can operate within a feed temperature range of 30 to 90 °C with alkali-treated CA. Following the alkali treatment, the CA membrane pores exhibit signs of collapse as a result of the deacetylation process. The alkaline treatment led to a marginal improvement in mechanical characteristics compared to the pristine CA membrane.

In conclusion, because of its resilience to changing salinity levels and possible integration with renewable energy sources, MD and PV show promise for unconventional desalination. However, wetting, fouling, and customisation issues necessitate more study and development. Beyond desalination, their special qualities make them appropriate for a variety of uses.

## Figures and Tables

**Table 1:** Composition of cellulosic membranes used.

Solvents (g)						
	Acetone	DMF	MA	G	DMP	D
CB	13	100	—	3		
CAB	10.5	74.5	10	—		
CA	40	21.5	8	5	12	62

**Table 2:** Properties of PTFE membranes.

Polymer	PTFE (0.1 µm)	PTFE (0.45 µm)
Support material	Laminated, PP non-woven	Laminated, PP netting
pH	1–14	1–14
Thickness	152–254 µm	64–127 µm
Water entry pressure	>4.1 bar (60 psi)	>0.76 bar (11 psi)
Application temperature range	82 °C	82 °C
Alcohol bubble point (MPa)	0.2–0.24	0.07–0.1
Alcohol flow rate (25 °C, Δp = −0.07 MPa) (mL/min/cm^2^)	5–10	25–40
Water contact angle	134 ± 3	127 ± 3

**Table 3:** The conditions and outcomes of each experiment performed for this study.

Membrane Type	NaCl Ci (g/L)	T_hot_ (°C)	T_cold_ (°C)	Operation Time (h)	Flux (kg/m^2^ h)	Salt Rejection (%)
CB	35	70	13	3.46	14.3	43.3
CAB	35	70	13	4.35	3.35	87.6
PTFE (0.1 µm)	35	80	13	08:25	9.98	99.89
	35	70	13	08:25	8.98	99.91
	35	60	13	08:25	5.78	98.99
	35	50	12	08:10	3.94	98.91
PTFE (0.45 µm)	35	70	12	07:50	13.35	99.86
	35	60	12	08:30	12.18	99.78
	96	60	13	06:40	8.88	99.83
	101.5	60	11	06:30	7.99	99.73
	138.8	60	12	06:43	6.23	99.68
	157	60	12	06:20	5.43	99.86
	157	60	10	07:30	5.62	99.89
CA	35	51	12	07:05	12.68	99.8
	35	62	12	07:10	15.74	99.81
	35	70	12	07:15	17.96	99.83
	35	80	12	07:12	18.24	99.98
	98	60	11	06:05	13.74	99.83
	135	60	12	05:55	12.87	99.83
	160	60	12	06:10	10.62	99.83
	160	60	9.5	07:10	11.08	99.88

**Table 5:** Performance evaluation of several MD membranes used to treat a brine solution.

Polymer	MD Module (Flat Sheet)	Thickness (nm)	Contact Angle (°)	LEP (kPa)	MD Configuration	Flux (kg/m^2^·h)	SR (%)	Time (h)	Ref.
PVDF	Single layer	~350	145.8	~52	DCMD	~58	99.9	65	[94]
PVDF/PSf	Dual layer	~710/800	140	~79	DCMD	~48	99.9	NA	[95]
PS	Single layer	~200	114	~150	DCMD	~31	99.9	10	[96]
PTFE	Hollow fibre	~210	158	~249	DCMD	~30	NA	8	[97]
PVDF	Single layer	~1150	139	~110	DCMD	~28	99.4	25	[98]
PVDF/MOFs	Single layer	~501	147.5	~41	DCMD	~27	NA	6	[99]
PAN/PVDF-HFP-PS/PDMS	Multi-layer	~266/531	148.5	~41	DCMD	~27	100	36	[100]
PVDF	Single layer	~350	124	~260	AGMD	~26	NA	30	[101]
PVDF-HFP/SiF	Single layer	~160	>90	~250	DCMD	~26	99.99	11	[102]
PEBAX/SiNPs	Single layer	~267	116.5	NA	VMD	~23	NA	14	[103]
PVDF-HFP/MOFs	Single layer	~293	134	~90	DCMD	~20	99.99	3	[104]
PVDF-HFP/N6	Dual layer	~240/125	126.3	~184	AGMD	~15	99	20	[105]
PVDF	Single layer	~378	139.6	~58	DCMD	~12	NA	20	[106]
SBS	Single layer	NA	132	NA	DCMD	~11	99.97	120	[107]
PTFE (0.45 µm)	Single layer	64,000–127,000	126	75	SGMD	13.35	99.86	8	This study
PTFE (0.1 µm)	Single layer	152,000–254,000	126	413	SGMD	8.98	99.91	8	This study

## Data Availability

The data presented in this study are available on request from the corresponding author.
